# Recent advances of biofuels and biochemicals production from sustainable resources using co-cultivation systems

**DOI:** 10.1186/s13068-019-1495-7

**Published:** 2019-06-22

**Authors:** Yujia Jiang, Ruofan Wu, Jie Zhou, Aiyong He, Jiaxing Xu, Fengxue Xin, Wenming Zhang, Jiangfeng Ma, Min Jiang, Weiliang Dong

**Affiliations:** 1 State Key Laboratory of Materials-Oriented Chemical Engineering, College of Biotechnology and Pharmaceutical Engineering, Nanjing Tech University, Puzhu South Road 30#, Nanjing, 211800 People’s Republic of China; 2 Jiangsu National Synergetic Innovation Center for Advanced Materials (SICAM), Nanjing Tech University, Nanjing, 211800 People’s Republic of China; 3 Jiangsu Key Laboratory for Biomass-based Energy and Enzyme Technology, Huaiyin Normal University, Huaian, 223300 People’s Republic of China

**Keywords:** Microbial consortia, Co-cultivation, Biofuels, Chemicals, Natural compounds, Sustainable resources

## Abstract

Microbial communities are ubiquitous in nature and exhibit several attractive features, such as sophisticated metabolic capabilities and strong environment robustness. Inspired by the advantages of natural microbial consortia, diverse artificial co-cultivation systems have been metabolically constructed for biofuels, chemicals and natural products production. In these co-cultivation systems, especially genetic engineering ones can reduce the metabolic burden caused by the complex of metabolic pathway through labor division, and improve the target product production significantly. This review summarized the most up-to-dated co-cultivation systems used for biofuels, chemicals and nature products production. In addition, major challenges associated with co-cultivation systems are also presented and discussed for meeting further industrial demands.

## Introduction

Pure cultures dominate the current industrial bioprocesses; however, they are confronted with challenges due to the increased requirement for higher efficiency of production and fulfillment of more complicated tasks. In nature, 99% microorganisms exist in the form of microbial consortia [1]. Inspired by the omnipresent natural microbial consortia, more attention has been paid on the bioprocess development of artificial ones, which pools different engineered microorganisms in one pot [2–4]. However, different from natural microbial communities, which exist mainly for the survival and growth in the environment, the artificial microbial consortia are specifically constructed to broaden the scope of feedstocks, enhance the productivity of target bio-products, etc. [5–7].

**Fig. 1:**
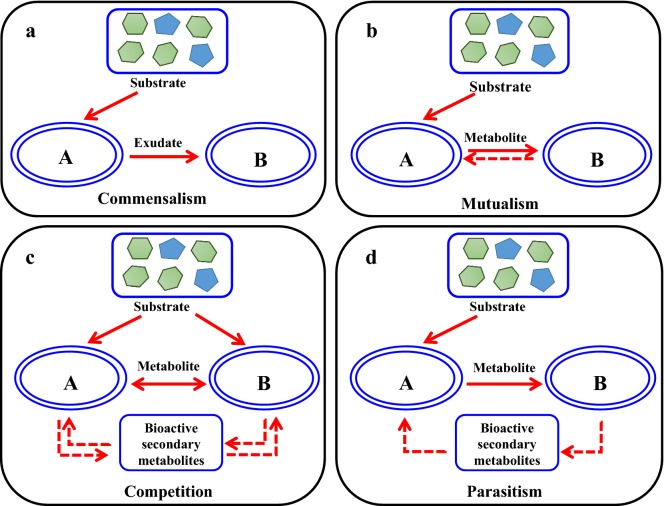
The schematic diagram for interaction modes of artificial microbial consortia. The interaction modes of artificial microbial consortia, including **a** commensalism, **b** mutualism, **c** competition and **d** parasitism

Diverse microbial communities within the same or different species have been set up to realize more complicated tasks [8–10]. In addition to treatment of wastewater, biodegradation of textile azo dye and dispose of contaminated soil, recently, co-cultivation systems were also applied to produce biofuels (bioethanol, biobutanol, biodiesel, etc.), bulk chemicals (lactic acid, 2-keto-l-gulonic acid, etc.) and natural products (alkaloids, polyketides, terpenes, flavonoid, etc.) [11–21]. These artificial microbial consortia interact mutually through the interaction of synergism, commensalism, competition, mutualism, etc. (Fig. 1) [1]. Elaboration of the underlying mechanism in microbial communities, such as the exchange of intermediate metabolites, cell-to-cell electrical connections, communications, etc. would guide the design of artificial microbial consortia and further improve the robustness and stability of the co-cultivation systems [22–25]. Accordingly, this review summarizes the superiority of co-cultivation systems compared with pure cultures and the most updated advances in artificial microbial consortia for the production of biofuels and chemicals from renewable sources. Nevertheless, further application and development of microbial consortia are still confronted with challenges, such as the uncharacterized microbial interaction mechanisms, etc.

### Advantages of co-cultivation systems over pure cultures

**Fig. 2:**
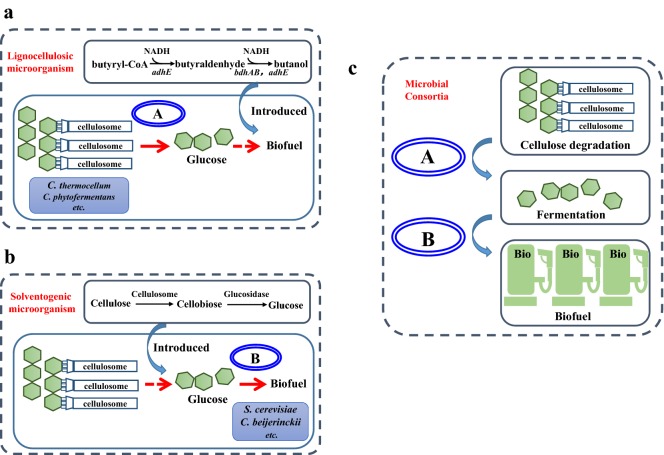
Comparison between pure cultures and microbial co-culturing systems for butanol production used lignocellulose. Two strategies for achievement of butanol production from lignocellulose via CBP. **a** the “native cellulolytic strategy”, in which butanol synthetic pathway was introduced into cellulolytic microorganism; **b** the “recombinant cellulolytic strategy”, in which cellulolytic enzymes were constructed into solventogenic ones. **c** The strategy for microbial co-culturing systems including lignocellulolytic microorganisms and solventogenic bacteria

Compared with pure cultures, co-cultivation systems could broaden the substrate utilization spectra. Lignocellulose is the most abundant sustainable recourses; however, due to the complexity of cellulose-degrading systems, single strain generally can not directly utilize it to synthesize valuable products [26, 27]. In general, two common strategies were developed: one is the incorporation of target product synthesis modules into cellulolytic microbes to achieve product generation from lignocellulose; the other is the introduction of cellulase systems into product-generating microbes (Fig. 2a, b) [28, 29]. However, the long and complex pathways including cellulase secretion and/or product synthesis would burden the metabolic stress and lead to low amounts of product generated [30, 31]. On the contrary, microbial consortia offer a simpler and more efficient approach to achieve this goal through the so-called consolidated bioprocessing (CBP), in which enzymes production, substrate hydrolysis and microbial fermentation are completed in one single reactor. For example, setting up co-cultivation systems including cellulolytic *Clostridium* sp. and non-cellulolytic *Thermoanaerobacter* sp. can achieve ethanol production from cellulose through CBP. Argyros et al. [32] set up an artificial *C. thermocellum*–*T. saccharolyticum* co-cultivation system, in which organic acids formation pathways were both removed in these two constituent strains. 38 g/L of ethanol was finally produced from 92 g/L of Avicel, which was approximately 80% theoretical maximum, indicating that *C. thermocellum* could be a cornerstone of a robust cellulolytic platform. On the other hand, the lagged utilization of pentose in both hexose and pentose mixtures is commonly found in most microbes, known as carbon catabolic repression (CCR), when bacteria are exposed to two or more carbon sources [33]. The sequential utilization of component sugars of lignocellulose materials would reduce the whole processes efficiency. Microbial consortia enable to rationally utilize different substrates based on the specific metabolic pathway. A novel binary culture can solve the problem flexibly, in which one could only consume glucose and the other could only consume xylose, shifting the interaction modes from the competition to the commensalism [34].

**Fig. 3:**
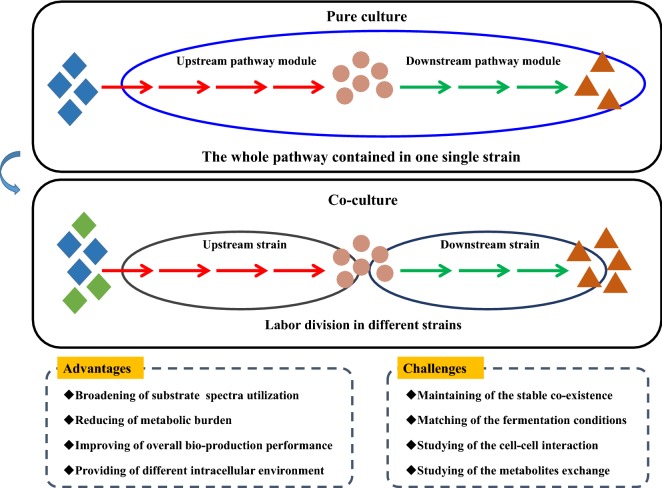
Illustration of the advantages and challenges of co-cultivation systems

When the biosynthetic pathway of target product is long and complicated, a large number of genes would be heterologously expressed in single strains. Generally, the biochemical properties and expression levels of introduced enzymes vary to a large extent. A single host cell cannot provide the optimal environment to perform the function well for all enzymes, while microbial consortia can provide diversified cellular environments for different enzymes. Especially, when a biosynthetic pathway is composed of both prokaryotic and eukaryotic enzymes, a combination of bacterial and fungal hosts would be highly advantageous over using either host alone [35]. In addition, excessive cellular resources consumption and overwhelming metabolic burden often lead to the impaired growth and/or poor biosynthetic behavior of single host strain [36]. Microbial consortia can reduce this metabolic burden through the strategy of labor division, which not only benefits the growth of individual strains, but also improves the performance of overall bio-production (Fig. 3) [37]. Furthermore, insufficient supply of precursors or excessive accumulation of intermediate products could both influence the end-products generation. In pure culture, the relative expression level of different genes is adjusted through promoter strength, gene copy number, ribosomal binding site etc. [38]. Building microbial consortia is a straightforward way to flexibly balance the biosynthetic strength through changing strain–strain ratios [39].

In pure cultures, most strains have individual suitable conditions for the growth. If cultural conditions changed, the growth and metabolism of strains would be affected. Microbial consortia could endure more changeable environments, providing an important new frontier for industrial production [1]. In microbial consortia, environmental disturbance can be dynamically balanced and regulated due to the coordination and cooperation of different strains. The undesired interference within different pathway modules in host strains would also be reduced [40]. Modular compartmentalization offers a new effective approach to limit negative interaction between pathway modules and improve the biosynthesis performance. Hence, microbial consortia commonly possess higher stability and robustness to environmental perturbations.

### Biofuels production by using co-cultivation systems

#### Bioethanol

As an environmentally friendly and sustainable source, biofuels production including bioethanol, biobutanol and biodiesel has gained considerable interests [41–43]. Bioethanol was regarded as one of the most promising biofuels, particularly as a carbon-neutral liquid transportation fuel [44]. Solventogenic yeasts, such as *Saccharomyces cerevisiae* and some bacteria, such as *Thermoanaerobacter* species are widely used to produce ethanol [45–47]. However, the feedstock spectrum is limited to some starchy-based materials [48]. Compared to grain-derived feedstocks, lignocellulose is a more economically feasible alternative because of its abundance and low cost [49, 50]. An artificial *Escherichia coli* binary culture was constructed for direct conversion of hemicellulose into ethanol. The final ethanol concentration reached 2.84 g/L, which is 55% of the theoretical yield [51]. In this binary system, one *E. coli* strain was engineered to hydrolyze hemicellulose to xylooligosaccharides through co-expression of two hemicellulase genes. Xylooligosaccharide-utilizing enzymes were then over-expressed in the other *E. coli* strain to realize the conversion of xylooligosaccharides into ethanol. This co-cultivation system distributed the metabolic burden through extracellular and intracellular expression of different functional enzymes, resulting in the improved ethanol production over pure cultures. Furthermore, cellulase system can also be built in a microbial consortium. For example, dual-microbe *Bacillus*/yeast system was developed for cellulosic ethanol production. Recombinant *B. subtilis* carries eight cellulosomal genes originating from *C. thermocellum*: one scaffolding protein gene (*cipA*), one cell-surface anchor gene (*sdbA*), two exo-glucosidase genes (*celK* and *celS*), two endo-glucanase genes (*celA* and *celR*), and two xylanase genes (*xynC* and *xynZ*). The partner *Kluyveromyces marxianus* KY3-NpaBGS carries a glucosidase (*NpaBGS*) gene from rumen fungus. Ultimately, 9.5 g/L of ethanol was produced from 20 g/L of cellulose (Table 1) [52].

**Table 1:** Biofuels and chemicals production by co-cultivation systems

Strains	Subtracts	Fermentation modes	Products	Titer	Time	References
*C. thermocellum*–*T. saccharolyticum*	92 g/L avicel	Batch	Ethanol	38 g/L	146 h	[32]
*E. coli* E609Y/pCRAXEXYL–*E. coli* KO11/pBBKXYN	10 g/L xylan	Batch	Ethanol	2.8 g/L	60 h	[51]
*C. thermocellum*–*K. marxianus*	20 g/L glucan	Batch	Ethanol	9.5 g/L	5 days	[52]
*C. thermocellum*–*Thermoanaerobacter* strains	20 g/L cellulose	Batch	Ethanol	6.6 g/L	~ 6 days	[56]
*C. phytofermentans*–*S. cerevisiae*	100 g/L cellulose	Batch	Ethanol	22 g/L	400 h	[57]
*C. thermocellum*–*C. beijerinckii*	88.9 g/L alkali extracted corn cobs	Batch	Butanol	10.9 g/L	200 h	[65]
*E. coli* strain BuT-3E–*E. coli* strain BuT-8L-ato	20 g/L glucose	Batch	Butanol	5.5 g/L	24 h	[67]
*Chlorella minutissima*–*A. awamori*	10 g/L glycerol	Batch	Palmitic (C16:0)	35.02 mg/L	–	[73]
*Chlorella minutissima*–*A. awamori*	10 g/L glycerol	Batch	Oleic (C18:1)	24.21 mg/L	–	[73]
*R. glutinis*–*Scenedesmus obliquus*	50 g/L glucose	Batch	Total lipid	~6 g/L	4 days	[74]
*T. reesei*–*L. pentosus*	50 g/L avicel	Batch	Lactate	34.7 g/L	215 h	[76]
*E. coli* ALS1073–*E. coli* ALS1074	22 g/L glucose + 33 g/L xylose	Batch	Lactate	37 g/L	24 h	[77]
*E. coli* P5.2–*E. coli* BC	20 g/L glycerol	Batch	Muconic acid	2 g/L	~ 48 h	[78]
*E. coli* P6.6–*E. coli* BXC	13.2 g/L glucose + 6.6 g/L xylose	Batch	Muconic acid	4.7 g/L	72 h	[34]
*G. oxydans*–*K. vulgare*	80 g/L d-sorbitol	Fed-batch	2-Keto-l-gulonic acid	76.6 g/L	36 h	[82]
*E. coli*–*S. cerevisiae*	Xylose	Fed-batch	Oxygenated taxanes	33 mg/L	120 h	[35]
*E. coli* C5–*E. coli* p168	20 g/L glycerol	Fed-batch	Flavan-3-ols	40.7 mg/L	54 h	[39]

Considering the complex of lignocellulose degradation enzymes, co-culturing cellulolytic microorganism with ethanol-producing one is a convenient and flexible approach to produce ethanol from lignocellulose through CBP. Cellulolytic *C. thermocellum* is a model organism for CBP; however, its application was limited due to the low ethanol yield [53–55]. Considering its efficient capability of cellulose degradation, *C. thermocellum* can be co-cultured with non-cellulolytic *Thermoanaerobacter* strains (X514 and 39E), which showed high efficiency of ethanol production [56]. The final ethanol production achieved at 7.56 and 6.59 g/L, respectively, which were significantly improved by 194–440%. The labor division is straightforward in this system: *C. thermocellum* is mainly responsible for cellulolysis, while *Thermoanaerobacter* sp. takes charge of ethanol production owing to its high-efficient ethanol production capability. The interaction within these two strains was through the exchange of intermediate metabolites. Similarly, a co-cultivation system, in which cellulose hydrolysis and ethanol production were conducted by *C. phytofermentans* and *S. cerevisiae,* was set up [57]. Glucosidase gene was overexpressed in *S. cerevisiae* to hydrolyze cellodextrin intracellularly. The connection of separated pathway modules was facilitated by the expression of intermediate cellodextrin transporters in the downstream *S. cerevisiae*. Finally, 22 g/L of ethanol was obtained from 100 g/L of cellulose using this artificial co-cultivation system.

#### Biobutanol

Biobutanol, a four-carbon and straight-chained alcohol is considered as more advanced biofuel over ethanol owing to its higher heating value, better inter-solubility, lower heat of vaporization, higher viscosity and lower corrosivity [58–61]. Generally, butanol was synthesized through traditional acetone–butanol–ethanol (ABE) fermentation process by solventogenic *Clostridium* sp. [62, 63]. However, most clostridia could not directly utilize polysaccharides, such as lignocellulose due to the inexpression of polysaccharide-degrading enzymes [64]. Hence, construction of microbial consortia may be an ideal strategy to achieve direct butanol production from renewable feedstocks (Fig. 2c). For example, a co-cultivation system composed of different solventogenic consortia (*C. thermocellum* ATCC 27405 and *C. beijerinckii* NCIMB 8052) was set up, which could directly produce butanol from lignocellulose [65]. The reducing sugars hydrolyzed by *C. thermocellum* ATCC 27405 were simultaneously metabolized by *C. beijerinckii* for butanol production. Meanwhile, the consumption of sugars could alleviate the feedback inhibition and further improve the degradation efficiency of alkali extracted corn cobs (AECC) by *C. thermocellum*. After optimization of cultivation temperature, 19.9 g/L of ABE (3.96 g/L of acetone, 10.9 g/L of butanol and 5.04 g/L of ethanol) were obtained from 88.9 g/L of AECC in 200 h, indicating the highest solvent production from lignocellulose through CBP (Table 1) [65]. Different from ethanol production, butanol synthetic pathway is more complex [66]. Introduction of butanol synthesis modules in model microorganisms, such as *E. coli,* would burden the metabolic stress. Whereas, dividing butanol biosynthetic pathway into butyrate-producing and butyrate-conversion modules in one co-culture system is more feasible. 5.5 g/L of butanol was finally produced in *E. coli*–*E. coli* system, which is twofold higher than that using pure culture [67]. Notably, volatile fatty acids travel freely across the cell membrane, which was recycled between the upstream and downstream *E. coli* strains to facilitate butyrate and butyryl-CoA inter-conversion.

#### Biodiesel

Biodiesel is another environmental-friendly biofuel, which can provide robust, massive, and enduring energy supply [68, 69]. Naturally, oleaginous algae are the well-known biodiesel producers [70]. However, several constraints hindered its further application. One major issue is the slow-growing rate and mutually incongruous nature of biomass and lipid accumulation [71, 72]. Co-cultivation of algae–fungus was proposed as an alternative approach for biodiesel production. An oleaginous fungus *Aspergillus awamori* was co-cultured with *Chlorella minutissima* MCC 27 and *C. minutissima* UTEX 2219, respectively. These two oleaginous algae–fungus consortia contain photoautotrophic green algae and obligate heterotrophic fungi. This system can utilize pure glycerol instead of glucose, which could reduce the production cost. A 2.6- and 3.9-fold increase in biomass and 3.4- and 5.1-fold increase in total lipid yields were observed in the co-cultures compared to the axenic cultures. Furthermore, C16:0 (31.26–35.02%) and C18:1 (21.14–24.21%) fatty acids were the major composites, suggesting that this co-culture system is a promising strategy for biodiesel production [73]. Microalgae are sunlight-driven cell factories that convert CO_2_ into lipids and O_2_ through the photosynthesis process. The production of O_2_ could further facilitate the growth of aerobic yeast, while the yeast mutually provides CO_2_ to the microalgae accompanied with the production of lipids. 40–50% of biomass and 60–70% of total lipids were increased compared to the single-culture batch [74]. The co-culture could provide the symbiotic environment for algae and yeast growth together, and the trace elements released through the natural lysis of the cells could be further utilized for the enhancement of cell growth. The co-culture of O_2_ provider *S. obliquus* and CO_2_ provider *R. glutinis* can offer gas transportation to both sides.

Taken together, microbial consortia can be constructed not only within the same species, but also in different genus, such as fungus–bacterium. Each member in microbial consortia interacts mutually through the exchange of metabolites. These microbial co-cultures provide the opportunity to achieve direct conversion of renewable sources into biofuel, maximization of substrate utilization rate, enhancement of yield and production, and reduction of process costs. However, as an immature but promising technology, application of microbial consortia for biofuel production at industrial scale still poses several challenges, such as the stability of microbial members in co-cultivation systems. More research efforts are still needed to develop more robust and stable microbial consortia that could be used for biofuels production.

### Bulk chemicals production by using co-cultivation systems

#### Lactic acid

**Fig. 4:**
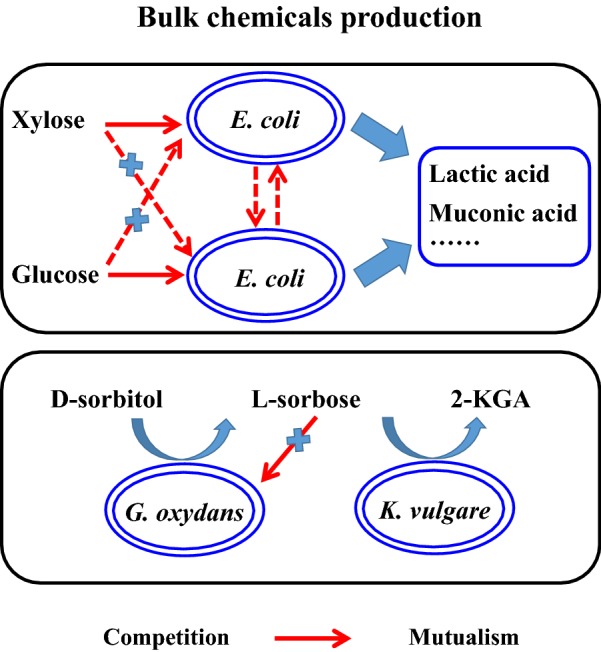
The strategy for improvement of bulk chemicals production by co-cultivation systems disengaged from competition interaction

In addition to biofuels, a wide range of bulk chemicals have also been produced using co-cultivation systems. Taking lactic acid, a versatile platform as an example, it is mainly produced from starchy-based materials or mono-sugars, which limits its large-scale production [75]. Recently, an artificial consortium composed of aerobic cellulolytic fungus *Trichoderma reesei* and lactic acid-producing bacterium *Lactobacilli pentosus* was metabolically constructed [76]. *T. reesei* acts as cellulose degraders, and *L. pentosus* is a robust lactic acid producer. The stable coexistence of these two strains is mainly based on the interaction of competitive cheater and cooperator. 34.7 g/L of lactic acid was produced from 5% (w/w) microcrystalline cellulose (Table 1). As mentioned above, CCR commonly occurs in most microbes when using lignocellulosic hydrolysate as the substrate. To overcome this obstacle, novel microbial consortia were constructed, in which one could only consume glucose and the other could only consume xylose (Fig. 4). The xylose-selective (glucose deficient) strain *E. coli* ALS1073 was constructed through the deletion of pyruvate formate lyase (*pflB*), glucokinase (*glk*), phosphotransferase system (*ptsG*), and IID^Man^ domain of the mannose PTS permease (*manZ*); while the glucose-selective (xylose deficient) strain *E. coli* ALS1074 has a *pflB* and xylose isomerase (*xylA*) deletion. The microbial consortium could simultaneously convert xylose and glucose into 37 g/L of lactate with a yield of 0.88 g/g [77]. In addition, the conversion rates of each sugar can be individually modulated to optimize the overall process.

#### Muconic acid

Muconic acid (MA) is another important bulk chemical; however, its production meets a challenge caused by the insufficient functional expression of enzymes due to the complex of synthesis pathway. Accordingly, an *E. coli*–*E. coli* binary consortium was constructed to achieve direct MA production from glycerol [78]. Two modules were constructed in different strains: the upstream strain *E. coli* P5.2 contained only the shikimate pathway ending with the synthesis of 3-dehydroshikimic acid (DHS); whereas *E. coli* BC was equipped with enzymes to assimilate and convert DHS into MA. To strengthen the penetration of the DHS into *E. coli* BC, ShiA permease, an endogenous *E. coli* membrane-bound transporter was overexpressed in strain BC under the control of a constitutive pyruvate decarboxylase promoter isolated from *Zymomonas mobilis*. Compared with the pure cultivation, co-cultivation can improve the production efficiency significantly. Finally, 2 g/L of MA with a yield of 0.1 g/g was produced in a batch bioreactor. This combination of pathway modularization and microbial co-cultivation shows strong potential for future metabolic engineering studies [78]. The bacterial consortium realized complex biosynthetic pathway engineering; however, the interaction within *E. coli*–*E. coli* is competition. Balancing the intermediate secretion and mixed sugars utilization could successfully overcome this limitation [34]. In this binary system, two *E. coli* strains were constructed individually to accommodate different pathway modules to reduce the metabolic stress in each strain. Effective regulation of the endogenous upstream pathway and expression of the challenging downstream heterologous enzymes were divided into two distinct cellular metabolic backgrounds, respectively. This *E. coli*–*E. coli* system also achieved simultaneous utilization of glucose and xylose (Fig. 4). Furthermore, a membrane-bound transporter was engineered to enhance the mass transfer of the pathway intermediate between the upstream and downstream strains. The microorganism consortium produced 4.7 g/L of MA with a yield of 0.35 g/g from glucose/xylose mixture, which is significantly higher than previous reports [34].

#### 2-Keto-l-gulonic acid

Currently, the most representative case for chemicals production using microbial consortia is 2-keto-l-gulonic acid (2-KGA), which is the precursor of vitamin C (L-ascorbic acid), an essential nutrient to maintain normal physiological activities in mammals. 110,000 tons of vitamin C is produced annually through bio-processes [79]. Currently, 2-KGA is mainly produced through two-step fermentation process, in which sorbitol is converted to sorbose by *Gluconobacter suboxydans* first, and then 2-KGA is synthesized from sorbose by co-cultivating with *B. megaterium* and *Ketogulonicigenium vulgare* [80, 81]. Recently, one step of 2-KGA production from d-sorbitol was developed (Fig. 4). In details, two sequential pathway modules were incorporated into *G. oxydans* and *K. vulgare* to achieve the conversion of D-sorbitol-to-sorbose and sorbose-to-2KGA, respectively, leading to a simplified one-step bioproduction process. *G. oxydans* was also metabolically engineered to reduce its competition against *K. vulgare* for sorbose. More importantly, the performance of this one-step process was comparable to the traditional two-step one with production and yield of 76.6 g/L and 89.7% within 36 h, respectively [82].

Not only limited to above-mentioned chemicals, co-cultivation systems are also applied for other bulk chemicals synthesis, such as succinic acid, butyric acid, etc. In construction of microbial consortia, the design of metabolic pathway is quite necessary, especially for the complex biosynthesis pathway to achieve labor division and reduce the metabolic stress. Engineering a membrane-bound transporter is also a rational way to enhance the mass transfer of the pivotal pathway intermediates between the upstream and downstream strains. In addition, reducing the competition interaction was also used in many co-cultivation systems, such as co-cultures of *E. coli* strains using different carbon source.

### Higher value-added chemicals production using co-cultivation systems

Natural products (NPs) are important sources for some novel bioactive compounds, such as drugs and other higher value-added compounds [13]. Typically, NPs can be extracted from plants and animals, but the low yield hinders their application. In addition, some bacteria and fungi are also important sources for NPs [83]. The most successful examples for NPs production using microbial consortia are taxol and flavonoids. Taxol is a well-known plant-derived terpenoids, because it is a chemotherapy medication used to treat various types of cancer [84–86]. The extracted yields of taxoids from the bark of the pacific yew tree (*Taxus brevifolia* Nutt.) were extremely low and limited. The biosynthesis of taxol involves at least 19 enzymatic steps starting from the universal diterpenoid precursor, geranylgeranyl diphosphate. The long and complicated pathway using pure culture would burden the metabolic capability, resulting in the production levels only maintained at μg/L levels [87, 88]. Division of synthetic pathway into different strains, such as bacterium–yeast strains would significantly improve the production level [37]. For example, *E. coli* can be engineered to use xylose as the substrate and overproduce taxadiene, which was the scaffold molecule of paclitaxel; *S. cerevisiae* was then engineered to express cytochrome P450s (CYPs) owing to its advanced protein expression machinery and abundant intracellular membranes, which functionalized taxadiene by catalyzing multiple oxygenation reactions. As known, *S. cerevisiae* is deficient in xylose utilization; hence, when xylose was used as the carbon source, *E. coli* would metabolize xylose to produce acetate and taxadiene first, and then acetate was used as the carbon source for *S. cerevisiae* growth. Accompanied with the consumption of acetate by *S. cerevisiae*, taxadiene could be further converted into taxol. The strategy of labor division in this system led to 33 mg/L of oxygenated taxanes including a monoacetylated dioxygenated taxane [35]. This success system shows an important advantage for designing the expression system and pathway in different strains, as they can be constructed and optimized in parallel to significantly improve the product titer. Furthermore, the system could combine dual properties of rapid production of taxadiene in *E. coli* with efficient oxygenation of taxadiene by *S. cerevisiae*.

Another typical example for NP production using microbial consortia is flavonoids, which also shows promising potential for pharmaceutical application [89]. The biosynthetic pathway from phenylpropanoic acids to flavan-3-ols was divided into the malonyl-CoA-dependent upstream module (phenylpropanoic acids to flavanones) and the NADPH-dependent downstream module (flavanones to flavan-3-ols). However, when this complicated pathway was expressed in pure cultures, flavan-3-ols titers from phenylpropanoic acids were very low. Chemler et al. [90] engineered *E. coli* binary system, which not only reduced the overwhelming metabolic burden, but also enabled to individually optimize the intermediate supply and co-factor provision in separate strains. After systematical process optimization, including carbon source, temperature, induction point, and inoculation ratio, 40.7 mg/L of flavan-3-ols was achieved with 970-fold flavonoids production improvement over the pure culture approach [39].

Except increasing the yields of previously described metabolites, microbial consortia can also induce new biosynthetic routes to bioactive metabolites [8, 91]. For example, new diorcinol J(1) was produced from a marine isolate of the fungi *Aspergillus sulphureus* KMM 4640 and *Isaria felina* KMM 4639 [92]. New lipoaminopeptides could be biosynthesized from two different fungi, *Mycogone rosea* and *Acremonium* sp.; however, the new derivatives were not detected in pure cultures of either fungus, suggesting that chimeric pathways resulting from co-culture can also lead to new natural products.

### Challenges and further perspectives for co-cultivation systems

Although many advantages existed for co-cultivation systems, advances and development of this emerging approach are still needed to address two critical challenges. One is how to maintain the stable co-existence of the constituent strains in the co-culture systems; the other is how to parallelly maintain the fermentation conditions, such as pH, temperature and oxygen supply. Different from natural microbial consortia existing for survival, the artificial co-cultures are constructed to optimize the production of target products. As such, the growth of involved co-culture members may be not compatible, often resulting in the competition for growth resources. In addition, the growth rates of microbial strains, especially different species vary to a large extent. As a result, co-cultivation of these species under a uniform growth condition can easily lead to the outgrowth of one specie over the other. Under such condition, adoption of microbial strains derived from the same species may be a better option. However, the general applicability of the same species is limited, as many biosynthesis processes require mixed biosynthesis capabilities from two or more different microbial species. Another alternative strategy is to engineer the co-culture members to grow and utilize separated carbon sources, reducing the growth competition and improving the growth compatibility. On the other hand, cooperative behavior must be robust to variations of environment, offering important insight for modular co-culture engineering design [93–95].

The design principles for microbial consortia are based on the interaction among microbial members, including cell–cell interaction, exchange of metabolites, etc. So far, most studies about microbial consortia mainly focused on the exchange of intermediate metabolites. However, due to the unknown genetic background of many wild-type species and uncharacterized microbial interaction mechanisms, the energy conversion efficiency of these microbial consortia was difficult to optimize, which greatly restricted their practical applications (Fig. 3). Except energy conversion, cell–cell interaction should also be emphasized [96]. For a desirable co-culturing system, positive interactions between two microorganisms are expected. The interactions between microorganisms in mixed culture environments may not always lead to desirable consequences. Hence, understanding the interactions between associated strains in artificial microbial consortia becomes more important. Synthetic biology tools, such as quorum sensing are being developed to manipulate the cell–cell interaction through signaling mechanisms, which shows great potential for growth and metabolic pathway coordination between the co-culture members in the future [97, 98]. In addition, building cross-feeding interactions within the microbial consortia is also an advantageous approach to connect cells and distribute metabolic functions [99]. Based on the understanding of the interaction among microbes, the robustness, stability and reproducibility could be further improved [100].

In addition, rationally designing parental strains through utilization of a combinatorial metabolic engineering approach for optimizing cellular phenotype would become future trends [101, 102]. Compartmentalization can effectively reduce the burden of fermentative strains, and microbial consortia could support plug-and-play biosynthesis of various target products. The co-culturing members can be engineered to specifically satisfy the need of the accommodated pathway modules, rather than the entire pathway. Also, the co-cultures can be easily programmed for new target biosynthetic pathways by re-organization or addition of the involved pathway modules/strains that have been pre-optimized for a specific part of the biosynthesis. A variety of products can be produced from the same upstream module by simply swapping the downstream modules. This intrinsic advantage of implementing modular design is well in line with the concept of modularity in synthetic biology and holds the potential of extensive applications in metabolic engineering.

## Conclusions

In recent years, construction of co-cultivation systems for biofuels and chemicals production has attracted more and more attention. Not only limited to simply mix the wild strains, co-cultivation has also expanded into synthetic biology. The introduction of synthetic intercellular communication into the cell engineering toolbox will open new frontiers and greatly contribute to the future success of synthetic biology and its applications. Although the production could be improved when using co-cultivation systems, challenges still exist. Currently, studies associated with co-cultivation systems are mainly constricted at the levels of exchange of intermediate metabolites. Other elements of environmental variation, such as energy flux, signal exchange and nutrient cycling are still unknown. Only based on the comprehensive understanding of the interaction among microbes, the improvement of robustness, stability and reproducibility can be further achieved.

## Data Availability

Not applicable.

## Abbreviations

CBP: consolidated bioprocessing
CCR: carbon catabolic repression
ABE: acetone–butanol–ethanol
AECC: alkali extracted corn cobs
MA: muconic acid
DHS: 3-dehydroshikimic acid
2-KGA: 2-keto-l-gulonic acid
NP: natural product

## References

[1] Ding MZ, Song H, Wang EX, Liu Y, Yuan YJ (2016). Design and construction of synthetic microbial consortia in China. Synth Syst Biotechnol.

[2] Jiang YJ, Zhang T, Lu JS, Dürre P, Zhang WM, Dong WL, Zhou J, Jiang M, Xin FX (2018). Microbial co-culturing systems: butanol production from organic wastes through consolidated bioprocessing. Appl Microbiol Biotechnol.

[3] Lindemann SR, Bernstein HC, Song HS, Fredrickson JK, Fields MW, Shou WY, Johnson DR, Beliaev AS (2016). Engineering microbial consortia for controllable outputs. ISME J.

[4] Minty JJ, Singer ME, Scholz SA, Bae CH, Ahn JH, Foster CE, Liao JC, Lin XN (2013). Design and characterization of synthetic fungal-bacterial consortia for direct production of isobutanol from cellulosic biomass. Proc Natl Acad Sci USA.

[5] Bhatia SK, Yoon JJ, Kim HJ, Hong JW, Hong YG, Song HS, Moon YM, Jeon JM, Kim YG, Yang YH (2018). Engineering of artificial microbial consortia of ralstonia eutropha and bacillus subtilis for poly (3-hydroxybutyrate-*co*-3-hydroxyvalerate) copolymer production from sugarcane sugar without precursor feeding. Bioresour Technol.

[6] Diender M, Stams AJM, Sousa DZ (2016). Production of medium-chain fatty acids and higher alcohols by a synthetic co-culture grown on carbon monoxide or syngas. Biotechnol Biofuels.

[7] Jiang LL, Liu HF, Mu Y, Sun YQ, Xiu ZL (2017). High tolerance to glycerol and high production of 1,3-propanediol in batch fermentations by microbial consortium from marine sludge. Eng Life Sci.

[8] Zuck KM, Shipley S, Newman DJ (2011). Induced production of n-formyl alkaloids from *Aspergillus fumigatus* by co-culture with *Streptomyces peucetius*. J Nat Prod.

[9] Dürre P (2016). Butanol formation from gaseous substrates. FEMS Microbiol Lett.

[10] Nakayama S, Kiyoshi K, Kadokura T, Nakazato A (2011). Butanol production from crystalline cellulose by co-cultured *Clostridium thermocellum* and *Clostridium saccharoperbutylacetonicum* N1-4. Appl Environ Microbiol.

[11] He F, Hu W, Li Y (2004). Biodegradation mechanisms and kinetics of azo dye 4BS by a microbial consortium. Chemosphere.

[12] Khouni I, Marrot B, Amar RB (2012). Treatment of reconstituted textile wastewater containing a reactive dye in an aerobic sequencing batch reactor using a novel bacterial consortium. Sep Purif Technol.

[13] Safonova E, Kvitko KV, Iankevitch MI, Surgko LF, Afti IA, Reisser W (2004). Biotreatment of industrial wastewater by selected algal-bacterial consortia. Eng Life Sci.

[14] Xu XH, Liu XM, Zhang L, Mu Y, Zhu XY, Fang JY, Li SP, Jiang JD (2018). Bioaugmentation of chlorothalonil-contaminated soil with hydrolytically or reductively dehalogenating strain and its effect on soil microbial community. J Hazard Mater.

[15] Sabra W, Dietz D, Tjahjasari D, Zeng AP (2010). Biosystems analysis and engineering of microbial consortia for industrial biotechnology. Eng Life Sci.

[16] Bertrand S, Bohni N, Schnee S, Schumpp O, Gindro K, Wolfender JL (2014). Metabolite induction via microorganism co-culture: a potential way to enhance chemical diversity for drug discovery. Biotechnol Adv.

[17] Eiteman MA, Lee SA, Altman R, Altman E (2009). A substrate-selective co-fermentation strategy with *Escherichia coli* produces lactate by simultaneously consuming xylose and glucose. Biotechnol Bioeng.

[18] Wang EX, Ding MZ, Ma Q, Dong XT, Yuan YJ (2016). Reorganization of a synthetic microbial consortium for one-step vitamin C fermentation. Microb Cell Fact.

[19] Schroeckh V, Scherlach K, Nützmann HW, Shelest E, Schmidtheck W, Schuemann J, Martin K, Hertweck C, Brakhage AA (2009). Intimate bacterial-fungal interaction triggers biosynthesis of archetypal polyketides in *Aspergillus nidulans*. Proc Natl Acad Sci USA.

[20] Xin FX, He JZ (2013). Characterization of a thermostable xylanase from a newly isolated *Kluyvera* species and its application for biobutanol production. Bioresour Technol.

[21] Tsai SL, Goyal G, Chen W (2010). Surface display of a functional minicellulosome by intracellular complementation using a synthetic yeast consortium and its application to cellulose hydrolysis and ethanol production. Appl Environ Microbiol.

[22] Lovley DR (2016). Happy together: microbial communities that hook up to swap electrons. ISME J.

[23] Fischbach MA, Segre JA (2016). Signaling in host-associated microbial communities. Cell.

[24] Kenny DJ, Balskus EP (2018). Engineering chemical interactions in microbial communities. Chem Soc Rev.

[25] Brenner K, You L, Arnold FH (2008). Engineering microbial consortia: a new frontier in synthetic biology. Trends Biotechnol.

[26] Deng AJ, Ren JL, Wang WJ, Li HL, Lin QX, Yan YH, Sun RC, Liu GL (2016). Production of xylo-sugars from corncob by oxalic acid-assisted ball milling and microwave-induced hydrothermal treatments. Ind. Crop. Prod..

[27] Li HL, Chen XF, Ren JL, Deng H, Peng F, Sun RC (2015). Functional relationship of furfural yields and the hemicellulose-derived sugars in the hydrolysates from corncob by microwave-assisted hydrothermal pretreatment. Biotechnol Biofuels.

[28] Olson DG, McBride JE, Shaw AJ, Lynd LR (2012). Recent progress in consolidated bioprocessing. Curr Opin Biotech.

[29] Yang X, Xu M, Yang ST (2015). Metabolic and process engineering of *Clostridium cellulovorans* for biofuel production from cellulose. Metab Eng.

[30] Shanmugam S, Hari A, Ulaganathan P, Yang F, Krishnaswamy S, Wu YR (2018). Potential of biohydrogen generation using the delignified lignocellulosic biomass by a newly identified thermostable laccase from *Trichoderma asperellum* strain BPLMBT1. Int J Hydrogen Energy.

[31] Mingardon F, Chanal A, Tardif C, Fierobe H (2011). The issue of secretion in heterologous expression of *Clostridium cellulolyticum* cellulase-encoding genes in *Clostridium acetobutylicum* ATCC 824. Appl Environ Microbiol.

[32] Argyros DA, Tripathi SA, Barrett TF, Rogers SR, Feinberg LF, Olson DG, Foden JM, Miller BB, Lynd LR, Hogsett DA (2011). High ethanol titers from cellulose by using metabolically engineered thermophilic, anaerobic microbes. Appl Environ Microbiol.

[33] Deutscher J (2008). The mechanisms of carbon catabolite repression in bacteria. Curr Opin Microbiol.

[34] Zhang HR, Pereira B, Li ZJ, Stephanopoulos G (2015). Engineering *Escherichia coli* co-culture systems for the production of biochemical products. Proc Natl Acad Sci USA.

[35] Zhou K, Qiao K, Steven E, Gregory S (2015). Distributing a metabolic pathway among a microbial consortium enhances production of natural products. Nat Biotechnol.

[36] Wu G, Yan Q, Jones JA, Tang YJ, Fong SS, Koffas MA (2016). Metabolic burden: cornerstones in synthetic biology and metabolic engineering applications. Trends Biotechnol.

[37] Agapakis CM, Boyle PM, Silver PA (2012). Natural strategies for the spatial optimization of metabolism in synthetic biology. Nat Chem Biol.

[38] Jones JA, Toparlak ÖD, Koffas MA (2015). Metabolic pathway balancing and its role in the production of biofuels and chemicals. Curr Opin Biotechnol.

[39] Jones JA, Koffas MA (2016). Optimizing metabolic pathways for the improved production of natural products. Methods Enzymol.

[40] Zhang H, Wang X (2016). Modular co-culture engineering, a new approach for metabolic engineering. Metab Eng.

[41] Jiang YJ, Xin FX, Lu JS, Dong WL, Zhang WM, Zhang M, Wu H, Ma JF, Jiang M (2017). State of the art review of biofuels production from lignocellulose by thermophilic bacteria. Bioresour Technol.

[42] Zhang J, Wang MY, Gao MT, Fang X, Yano S, Qin SL, Xia RR (2013). Efficient acetone–butanol–ethanol production from corncob with a new pretreatment technology—wet disk milling. Bioenerg Res..

[43] Karmee SK, Lin CSK (2014). Valorisation of food waste to biofuel: current trends and technological challenges. Sustain Chem Process.

[44] Carroll A, Somerville C (2009). Cellulosic biofuels. Annu Rev Plant Biol.

[45] Balat M (2011). Production of bioethanol from lignocellulosic materials via the biochemical pathway: a review. Energy Convers Manage.

[46] Shaw AJ, Covalla SF, Miller BB, Firliet BT, Hogsett DA, Herring CD (2012). Urease expression in a *Thermoanaerobacterium saccharolyticum* ethanologen allows high titer ethanol production. Metab Eng.

[47] Hon S, Olson DG, Holwerda EK, Lanahan AA, Murphy JL, Maloney MI, Zheng TY, Papanek B, Guss AM, Lynd LR (2017). The ethanol pathway from *Thermoanaerobacterium saccharolyticum* improves ethanol production in *Clostridium thermocellum*. Metab Eng.

[48] Dürre P, Richard T (2011). Microbial energy conversion revisited. Curr Opin Biotechnol.

[49] Jiang YJ, Guo D, Lu JS, Dürre P, Dong WL, Yan W, Zhang WM, Ma JF, Jiang M, Xin FX (2018). Consolidated bioprocessing of butanol production from xylan by a thermophilic and butanologenic *Thermoanaerobacterium* sp. M5. Biotechnol Biofuels.

[50] Saggi SK, Dey P (2016). An overview of simultaneous saccharification and fermentation of starchy and lignocellulosic biomass for bio-ethanol production. Biofuels.

[51] Shin HD, Mcclendon S, Vo T, Chen RR (2010). *Escherichia coli* binary culture engineered for direct fermentation of hemicellulose to a biofuel. Appl Environ Microbiol.

[52] Ho CY, Chang JJ, Lee SC, Chin TY, Shih MC, Li WH, Huang CC (2012). Development of cellulosic ethanol production process via, co-culturing of artificial cellulosomal *Bacillus* and kefir yeast. Appl Energy.

[53] Demain AL, Newcomb M, Wu JHD (2005). Cellulase, clostridia, and ethanol. Microbiol Mol Biol Rev.

[54] Beguin P, Aubert JP (1994). The biological degradation of cellulose. FEMS Microbiol Rev.

[55] Lynd LR (1989). Production of ethanol from lignocellulosic materials using thermophilic bacteria: critical evaluation of potential and review. Adv Biochem Eng Biotechnol.

[56] He Q, Hemme CL, Jiang H, He Z, Zhou J (2011). Mechanisms of enhanced cellulosic bioethanol fermentation by co-cultivation of *Clostridium* and *Thermoanaerobacter* spp. Bioresour Technol.

[57] Zuroff TR, Xiques SB, Curtis WR (2013). Consortia-mediated bioprocessing of cellulose to ethanol with a symbiotic *Clostridium phytofermentans*/yeast co-culture. Biotechnol Biofuels.

[58] Jin C, Yao M, Liu H, Lee CFF, Ji J (2011). Progress in the production and application of n-butanol as a biofuel. Renew Sust Energ Rev.

[59] Zheng T, Olson DG, Tian L, Bomble YJ, Himmel ME, Lo J, Hon S, Shaw AJ, van Dijken JP, Lynd LR (2015). Cofactor specificity of the bifunctional alcohol and aldehyde dehydrogenase (AdhE) in wild-type and mutant *Clostridium thermocellum* and *Thermoanaerobacterium saccharolyticum*. J Bacteriol.

[60] Trindade WRDS, Santos RGD (2017). Review on the characteristics of butanol, its production and use as fuel in internal combustion engines. Renew Sust Energ Rev.

[61] Nguyen S, Ala F, Cardwell C, Cai D, McKindles KM, Lotvola A, Hodges S, Deng Y, Tiquia-Arashiro SM (2013). Isolation and screening of carboxydotrophs isolated from composts and their potential for butanol synthesis. Environ Technol.

[62] Sun C, Zhang S, Xin FX, Shanmugam S, Wu YR (2018). Genomic comparison of *Clostridium* species with the potential of utilizing red algal biomass for biobutanol production. Biotechnol Biofuels.

[63] Shanmugam S, Sun C, Zeng X, Wu YR (2018). High-efficient production of biobutanol by a novel *Clostridium* sp. strain WST with uncontrolled pH strategy. Bioresour Technol.

[64] Jiang YJ, Chen TP, Dong WL, Zhang M, Zhang WM, Wu H, Ma JF, Jiang M, Xin FX (2018). The draft genome sequence of *Clostridium beijerinckii* NJP7, a unique bacterium capable of producing isopropanol-butanol from hemicellulose through consolidated bioprocessing. Curr Microbiol.

[65] Wen ZQ, Wu MB, Lin YJ, Yang LR, Lin JP, Cen PL (2014). A novel strategy for sequential co-culture of *Clostridium thermocellum* and *Clostridium beijerinckii*, to produce solvents from alkali extracted corn cobs. Process Biochem.

[66] Pfromm PH, Amanor-Boadu V, Nelson R, Vadlani P, Madl R (2010). Bio-butanol vs. bio-ethanol: a technical and economic assessment for corn and switchgrass fermented by yeast or *Clostridium acetobutylicum*. Biomass Bioenerg.

[67] Saini M, Chen MH, Chiang CJ, Chao YP (2015). Potential production platform of n-butanol in *Escherichia coli*. Metab Eng.

[68] Lai EPC (2014). Biodiesel: environmental friendly alternative to petrodiesel. J Pet Environ. Biotechnol..

[69] Mata TM, Martins AA, Caetano NS (2010). Microalgae for biodiesel production and other applications: a review. Renew Sust Energ Rev.

[70] Singh A, Olsen SI (2011). A critical review of biochemical conversion, sustainability and life cycle assessment of algal biofuels. Appl. Energ..

[71] Sharma KK, Schuhmann H, Schenk PM (2012). High lipid induction in microalgae for biodiesel production. Energies.

[72] Li Y, Han D, Sommerfeld M, Hu Q (2011). Photosynthetic carbon partitioning and lipid production in the oleaginous microalga *Pseudochlorococcum* sp. (Chlorophyceae) under nitrogen-limited conditions. Bioresour Technol.

[73] Dash A, Banerjee R (2017). Enhanced biodiesel production through phyco-myco co-cultivation of *Chlorella minutissima* and *Aspergillus awamori*: an integrated approach. Bioresour Technol.

[74] Yen HW, Chen PW, Chen LJ (2015). The synergistic effects for the co-cultivation of oleaginous yeast-*Rhodotorula glutinis* and microalgae-*Scenedesmus obliquus* on the biomass and total lipids accumulation. Bioresour Technol.

[75] Abdel-Rahman MA, Tashiro Y, Sonomoto K (2013). Recent advances in lactic acid production by microbial fermentation processes. Biotechnol Adv.

[76] Shahab RL, Luterbacher JS, Brethauer S, Studer MH (2018). Consolidated bioprocessing of lignocellulosic biomass to lactic acid by a synthetic fungal-bacterial consortium. Biotechnol Bioeng.

[77] Eiteman MA, Lee SA, Altman R, Altman E (2009). A substrate-selective co-fermentation strategy with *Escherichia coli* produces lactate by simultaneously consuming xylose and glucose. Biotechnol Bioeng.

[78] Zhang HR, Li ZJ, Pereira B, Stephanopoulos G (2015). Engineering *E. coli*–*E. coli*, co-cultures for production of muconic acid from glycerol. Microb Cell Fact.

[79] Bremus C, Herrmann U, Bringer-Meyer S, Sahm H (2006). The use of microorganisms in l-ascorbic acid production. J Biotechnol.

[80] Zhu Y, Liu J, Liu J, Du G, Zhou J, Chen J (2012). A high throughput method to screen companion bacterium for 2-keto-l-gulonic acid biosynthesis by co-culturing *Ketogulonicigenium vulgare*. Process Biochem.

[81] Zhang J, Liu J, Shi Z, Liu L, Chen J (2010). Manipulation of *B. megaterium* growth for efficient 2-KLG production by *K. vulgare*. Process Biochem.

[82] Wang EX, Ding MZ, Ma Q, Dong XT, Yuan YJ (2016). Reorganization of a synthetic microbial consortium for one-step vitamin C fermentation. Microb Cell Fact.

[83] Cragg GM, Newman DJ (2013). Natural products: a continuing source of novel drug leads. Biochim Biophys Acta.

[84] Hainsworth JD, Thompson DS, Greco FA (1995). Paclitaxel by 1-hour infusion: an active drug in metastatic non-small-cell lung cancer. J Clin Oncol.

[85] Suffness M, Wall ME, Suffness M (1995). Discovery and development of Taxol. Taxol science and applications.

[86] Kingston DGI (2007). The shape of the things to come: structural and synthetic studies of Taxol and related compounds. Phytochemistry.

[87] Engels B, Dahm P, Jennewein S (2008). Metabolic engineering of taxadiene biosynthesis in yeast as a first step towards taxol (paclitaxel) production. Metab Eng.

[88] Peng Z, Zhou PP, Yu LJ (2009). An endophytic taxol-producing fungus from taxus media, *Cladosporium cladosporioides* MD2. Curr Microbiol.

[89] Hooper L, Kay C, Abdelhamid A, Kroon PA, Cohn JS, Rimm EB, Cassidy A (2012). Effects of chocolate, cocoa, and flavan-3-ols on cardiovascular health: a systematic review and meta-analysis of randomized trials. Am J Clin Nutr.

[90] Chemler JA, Lock LT, Koffas MAG, Tzanakakis ES (2007). Standardized bio-synthesis of flavan-3-ols with effects on pancreatic beta-cell insulin secretion. Appl Microbiol Biotechnol.

[91] Ola ARB, Thomy D, Lai D, Brötzoesterhelt H, Proksch P (2013). Inducing secondary metabolite production by the endophytic fungus *Fusarium tricinctum* through co-culture with *Bacillus subtilis*. J Nat Prod.

[92] Zhuravleva OI, Kirichuk NN, Denisenko VA, Dmitrenok PS, Yurchenko EA, Min′Ko EM, Ivanets EV, Sh Afiyatullov Sh (2016). New diorcinol J produced by co-cultivation of marine fungi *Aspergillus sulphureus* and *Isaria feline*. Chem Nat Compd.

[93] Jagmann N, Philipp B (2014). Design of synthetic microbial communities for biotechnological production processes. J Biotechnol.

[94] Song H, Ding MZ, Jia XQ, Ma Q, Yuan YJ (2014). Synthetic microbial consortia: from systematic analysis to construction and applications. Chem Soc Rev.

[95] Wintermute EH, Silver PA (2010). Emergent cooperation in microbial metabolism. Mol Syst Biol.

[96] Shong J, Diaz MRJ, Collins CH (2012). Towards synthetic microbial consortia for bioprocessing. Curr Opin Biotechnol.

[97] Schertzer JW, Boulette ML, Whiteley M (2009). More than a signal: non-signaling properties of quorum sensing molecules. Trends Microbiol.

[98] Choudhary S, Schmidt-Dannert C (2010). Applications of quorum sensing in biotechnology. Appl Microbiol Biotechnol.

[99] Pande S, Shitut S, Freund L, Westermann M, Bertels F, Colesie C, Bischofs IB, Kost C (2015). Metabolic cross-feeding via intercellular nanotubes among bacteria. Nat Commun..

[100] Fazzini RAB, Preto MJ, Quintas ACP, Bielecka A, Timmis KN, Dos Santos VAPM (2010). Consortia modulation of the stress response: proteomic analysis of single strain versus mixed culture. Environ Microbiol.

[101] Santos CN, Xiao W, Stephanopoulos G (2012). Rational, combinatorial, and genomic approaches for engineering l-tyrosine production in *Escherichia coli*. Proc Natl Acad Sci USA.

[102] Xie ZX, Li BZ, Mitchell LA, Wu Y, Qi X, Jin Z (2017). “perfect” designer chromosome v and behavior of a ring derivative. Science.

